# Scaling up antiretroviral treatment and improving patient retention in care: lessons from Ethiopia, 2005-2013

**DOI:** 10.1186/1744-8603-10-43

**Published:** 2014-05-27

**Authors:** Yibeltal Assefa, Achamyeleh Alebachew, Meskele Lera, Lut Lynen, Edwin Wouters, Wim Van Damme

**Affiliations:** 1 Ethiopian Public Health Institute, Addis Ababa, Ethiopia; 2 Federal HIV/AIDS Prevention and Control Office, Addis Ababa, Ethiopia; 3 Department of Clinical Sciences, Institute of Tropical Medicine, Antwerp, Belgium; 4 Department of Social Sciences, Antwerp University, Antwerp, Belgium; 5 Department of Public Health, Institute of Tropical Medicine, Antwerp, Belgium; 6 School of Public Health, University of the Western Cape, Cape Town, South Africa

**Keywords:** Antiretroviral treatment, Scaling up, Retention in care, Resource-limited settings

## Abstract

**Background:**

Antiretroviral treatment (ART) was provided to more than nine million people by the end of 2012. Although ART programs in resource-limited settings have expanded treatment, inadequate retention in care has been a challenge. Ethiopia has been scaling up ART and improving retention (defined as continuous engagement of patients in care) in care. We aimed to analyze the ART program in Ethiopia.

**Methods:**

A mix of quantitative and qualitative methods was used. Routine ART program data was used to study ART scale up and patient retention in care. In-depth interviews and focus group discussions were conducted with program managers.

**Results:**

The number of people receiving ART in Ethiopia increased from less than 9,000 in 2005 to more than 439, 000 in 2013. Initially, the public health approach, health system strengthening, community mobilization and provision of care and support services allowed scaling up of ART services. While ART was being scaled up, retention was recognized to be insufficient. To improve retention, a second wave of interventions, related to programmatic, structural, socio-cultural, and patient information systems, have been implemented. Retention rate increased from 77% in 2004/5 to 92% in 2012/13.

**Conclusion:**

Ethiopia has been able to scale up ART and improve retention in care in spite of its limited resources. This has been possible due to interventions by the ART program, supported by health systems strengthening, community-based organizations and the communities themselves. ART programs in resource-limited settings need to put in place similar measures to scale up ART and retain patients in care.

## Introduction

The advent of potent antiretroviral treatment (ART) has dramatically reduced rates of mortality and morbidity, improved quality of life, revitalized communities and transformed perceptions on HIV/AIDS from a plague to a manageable chronic illness [1-4]. Cognizant of these benefits, countries have been expanding access to ART [5]. The rapid expansion of ART is one of the most remarkable achievements in recent public health history. Enormous progress has been made in the last eight years with the scale-up of ART in low- and middle income countries. More people initiated ART in recent years than in previous years, with the number of people living with HIV receiving treatment rising by more than 20% in 2012 compared to 2011 [5]. As a result, ART was provided to more than nine million people by the end of 2012, which is a 22-fold increase since 2003 [6]. The World Health Organization (WHO) has recently recommended initiating ART with higher CD4-cells count, up to 500 per micro-liter of blood [7]. This is a call for countries to identify context specific options for increasing access and utilization of ART. Although ART programs in resource-limited settings have expanded treatment access and reached millions of PLwHA, inadequate retention in care has been a real challenge for the programs and has undermined efforts to optimize patient and program outcomes [8,9]. Therefore, ensuring good retention in care remains one of the key challenges for ART programs while the treatment should still be scaled up further based on the 2013 WHO guidelines [7,9].

Ethiopia has been scaling up ART towards universal access [10-12]. Retention in care was identified as a challenge for the program while the treatment was being scaled up [11]. The country has hence started implementing a variety of approaches to improve retention in care. It is therefore important that the lessons learnt in scaling up ART and retaining patients in care, during the past decade, are systematically documented in such a way that these lessons will be valuable for scaling up ART further and improving patient retention. In accordance with the above-described research needs, the objectives of this study were: (1) to describe the progress in scaling up ART and retaining patients in care in Ethiopia, (2) to identify the approaches employed to scale up ART in Ethiopia, and (3) to identify the interventions that have been implemented to improve retention in care in Ethiopia.

## Methods

### Setting: HIV/AIDS and ART program in Ethiopia

The HIV/AIDS epidemic has remained one of the important public health challenges in Ethiopia since it was first recognized in 1984. The epidemic has passed through various stages, from a concentrated epidemic among key populations (such as commercial sex workers and long distance truck drivers) over a phase of rapid transmission to the current generalized and fairly declining phase with a drop in HIV prevalence from 5.5% to 2.1% between 2003 and 2012 among pregnant women attending antenatal care [12-14]. Prevalence has remained markedly higher in urban areas (1.9%) throughout the epidemic period, while the rural areas have a very low prevalence (0.9%). This has been possible due to the development and implementation of policies, strategic plans and implementation guidelines necessary to effectively guide the prevention and control efforts. Moreover, the intensity and quality of interventions against HIV/AIDS have shown consistent and substantial improvements during the same period [12-14].

A fee-based ART program was officially started in Ethiopia in 2003. A number of initiatives, including the Global Fund, PEPFAR and others, have enabled the rapid expansion of free ART in the country [12]. Subsequently, the country launched Millennium AIDS Campaign (MAC-E) in November 2006 (at the eve of the new Ethiopian Millennium) with the theme of “Speed, Volume, and Quality”. The MAC-E, which was run in three phases, resulted in quantum leaps in the uptake of HIV testing and counseling (HTC) and ART services. More than 1.6 million people were tested for HIV and 42,859 patients were initiated on ART during the two phases of the MAC-E. The last phase of MAC-E resorted into regular service delivery mode by implementing an accelerated plan for comprehensive HIV/AIDS services in order to maintain campaign achievements [12].

The first-line ART regimen consists of two nucleoside reverse-transcriptase inhibitors plus one non-nucleoside reverse-transcriptase inhibitor. The currently recommended first-line regimens, in decreasing order of preference, are: TDF-3TC-EFV, or TDF-3TC-NVP, or AZT-3TC-EFV, or AZT-3TC-NVP. Patients are started on ART based on clinical and/or immunological criteria [13].

The eligibility criterion for ART has changed over the years. Until the end of 2012, the eligibility criterium for initiating ART was CD4-cells count less than 200 cells per micro-liter of blood, or WHO stage 4. Currently, all patients with CD4-cells count less than 350 cells per micro-liter of blood, or patients with WHO stages 3 and 4 are eligible for ART initiation. Once patients are started on ART, they are advised to visit the clinic for follow up (including CD4, hematology and chemistry). This will be done every two weeks during the first month, every month until the end of the third month, and then every three months unless they have problems [13].

### Study design

A mix of quantitative and qualitative methods was used to achieve the objectives of the study. The quantitative method included a review and analysis of routine program data reported from all health facilities to the Federal Ministry of Health and its Agencies. It was used to describe the progress in scaling up ART and retaining patients in care in Ethiopia. The qualitative methods, including both in-depth interviews and focus group discussions (FGDs), were conducted to: (1) identify the approaches employed to scale up ART, and (2) find out the interventions implemented to improve retention in care in Ethiopia.

In-depth interviews (with 72 key informants) and FGDs (two with 6 key informants each) were conducted with key informants including program managers from both government and development partners from July to September 2013. The participants were purposively selected as key informants to generate rich, relevant and diverse information pertinent to ART scale up and retention in care. The interviews and discussions were conducted in local language (Amharic) and tape recorded. Question guides were used to facilitate the interviews and discussions.

### Data analysis

The quantitative data on ART scale up and retention in care were entered, cleaned and analyzed using Excel. The data on retention in care was analyzed using EPI-Info 2005 version 3.3.2 statistical software. Constant comparison was conducted on the field notes and transcripts of the records of the qualitative data, based on the question guides for the interviews and FGDs, to develop themes around the interventions implemented to scale up ART and retain patients in care.

### Ethics Statement

This study was approved by the ethical clearance committee of the Ethiopian Health and Nutrition Research Institute. We obtained informed verbal-consent from study participants for conducting and recording the interviews. The verbal consent was tape recorded in local language.

## Results

### Scaling up ART

**Figure 1:**
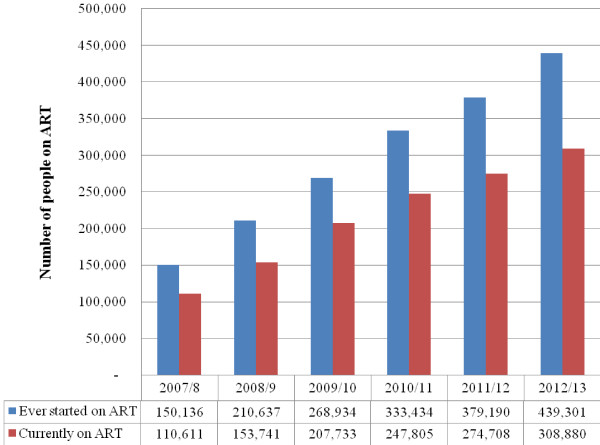
Trend in number of people receiving antiretroviral treatment services in Ethiopia, 2007/8-2012/13.

By mid 2013, over 913 health facilities (including 143 hospitals and 770 health centers) were providing ART nationwide [14]. The number of patients who had been ever started (ES) on ART increased from 9,000 at the beginning of 2005 to more than 439,000 by mid-2013 [14]. There were 308,880 patients (with 75% ART coverage) who are currently on ART (COA) by mid-2013 (Figure 1) [14].

The proportion of patients receiving ART outside Addis Ababa increased from 35% in 2005 to 77% in 2013. The proportion of patients who received ART in primary health care facilities had increased from less than 2% in 2006 to more than 25% in 2013 [12,14]. One of the achievements of the large scale free ART program was its increased utilization (initiation on ART) by women. When free ART program was first introduced in Ethiopia in 2005, women had substantially lower utilization (25%) than men (75%). Currently, more women (55%) than men (45%) are on treatment [12,14,15].

This large scale expansion of the ART program has been possible because of a number of initiatives in the country. The most important ones identified by our study were (1) the implementation of the public health approach for ART delivery, (2) expansion of HIV testing and counseling (HTC), (3) mobilization of the community, (4) strengthening of the health systems, and (5) provision of care and support services by the ART program and community-based organizations.

### The public health approach

The public health approach, including task shifting and decentralization, has been instrumental in the large scale expansion of ART in the country [11,12,16]. Health officers and nurses are initiating patients on ART in Ethiopia. Community health workers and expert patients are doing adherence counseling and defaulter tracing activities [17]. ART services have been decentralized to primary health centers. The number of primary health centers providing ART increased from nil in 2005 to more than 650 in 2013 [17]. Task shifting and decentralization were possible due to standardization of treatment regimens, training of providers and simplification of laboratory and patient monitoring mechanisms [12].

### Expanding access to and utilization of HIV Testing and Counseling services

**Figure 2:**
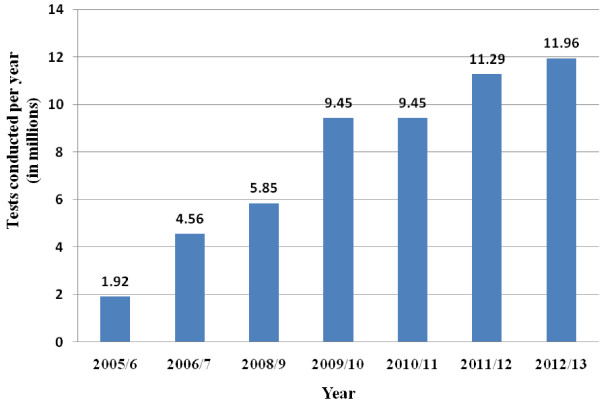
Number of people tested for HIV in Ethiopia, 2005/6-2012/13.

HTC is an important entry for accessing care and treatment services. A variety of approaches have been utilized to increase the number of people tested for HIV. These include both facility-and community-based, and opt-out and opt-in approaches. Community–based approaches (both mobile and outreach) have been essential in increasing awareness and access to HCT. The number of health facilities providing HTC services increased from 568 in 2005/6 to 3,014 in 2012/13 [14]. The number of people tested for HIV also increased from less than two million to 12 million per year during the same period (Figure 2).

One of the key informants emphasized that linkage to care was a challenge once people are tested positive for HIV. He said,

“When HTC service was being scaled up, it was recognized that linkage to care after HIV testing was indeed very low (around 61%). HIV testing is a means (point of entry) not an end by itself. The program has to make sure that people tested positive have adequate care and support services. The country has thus developed a strategic framework for improving linkage to care.”

### Community mobilization

Community mobilization for treatment literacy, stigma reduction and care and support services has been instrumental in increasing utilization of ART and improving retention in care. Community conversation (CC), which is a government initiative owned and led by the community, is one of the approaches which have been implemented to increase awareness and improve services utilization. It has been widely implemented throughout the country since 2004. It has basically two types: general CC and targeted CC. The general CC is for all members of the community. The targeted CC is for people living with HIV/AIDS and most-at-risk populations. Sixty individuals together form one community conversation group. They meet every 15 days for 10 months to complete one cycle. The group discusses social, cultural, economic and behavioral issues which are believed to impede the prevention and control of HIV/AIDS [14]. The group then develops an action plan to address these problems. The community uses its own resources to implement these activities [14]. Community opinion leaders (priests, sheiks, kebele administrators, and elders) are role models to be emulated by fellow members of the community. Currently, CC is institutionalized with the health extension program (a community health program focusing on primary health care provision at community level) and is facilitated by the health extension worker (female community health worker running the community health services). It is also addressing issues beyond HIV/AIDS, such as malaria, sanitation and nutrition [14].

### Health systems strengthening

The country has been investing a lot in health systems strengthening. Our key informants said:

“The rapid expansion of ART couldn’t have been possible without the investment in health systems. A lot of health centers have been constructed, the logistics management systems has been upgraded and the laboratory system has also been strengthened so that ART will be available in both rural and urban settings.”

### Infrastructure

A health facility expansion strategy was articulated in 2004. As a result, a total of 1,324 health centers were constructed by the Federal Ministry of Health. Out of these, 1,076 health centers were constructed through the support of the Global Fund. The remaining are constructed through the support from the Global Alliance for Vaccines and Immunization (155 HCs) and the Millennium Development Goals (MDGs) pool fund (93 HCs) [18]. An equivalent number of health centers were also constructed by domestic funding. As a result, the number of health centers in the country has increased from 668 in 2007 to 3,245 in 2013.

### Supply chain management

The Ethiopian Pharmaceutical Fund and Supply Agency was established in 2007 to strengthen the supply chain management system for health commodities. One of the key informants said:

*“The agency conducts national quantification exercises for antiretrovirals and other health commodities annually. The agency is improving its distribution capacity by constructing new modern warehouses across the country. It will have a total number of 17 new branches in the near future. The storage capacity will thus be increased from 45,000 m*^*3*^*to 580,000 m*^*3*^*. The number of vehicles has increased from 21 to 171 in five-years time. The agency is also currently working to fully automate its central and branch warehouse management systems.”*

Most of the funding for the agency comes from the Global Fund and PEPFAR support. The agency also generates revenue for its procurement, storage and distribution services [19].

### Laboratory

Laboratory services are essential components of the health care system. The Ethiopian Health and Nutrition Research Institute (EHNRI) was mandated by the Federal Ministry of Health to strengthen the national laboratory system in October 2005. EHNRI prepared the first Laboratory Master Plan in 2005 with the focus on building the HIV laboratory system in the country. Significant achievements have been made in expanding the HIV laboratory services, including CD4-cells count monitoring machines which have increased from 20 to 200 between 2005 and 2013. The national laboratory has also increased its capacity for external quality assurance, equipment maintenance and sample referral systems. The sample referral system has also played a crucial role in transporting samples to and from health facilities to conduct laboratory tests for treatment initiation and monitoring. The sample referral system is designed to support health facilities which do not have CD4 and other related laboratory services. This has been possible due to the support from both PEPFAR and the Global Fund [20].

### Human resources for health

The Ministry of Health of Ethiopia understood that human resources for health (HRH) was an important bottleneck for expanding access to health services in the country. Hence, the Ministry has designed a ‘flooding strategy’ which aimed to increase the number and type of HRH massively. There were thus major investments in universities and other training institutions to train different health cadres including physicians. As a result, the number of qualified health staff, including nurses, medical doctors, pharmacists, and health extension workers increased from less 46,000 in 2002 to over 70,000 in 2013 [21]. It is believed that the flooding strategy, together with the task shifting approach, has enabled the ART program to increase access to and utilization of ART in Ethiopia.

### Provision of care and support services

Provision of care and support services is one of the strategies utilized to increase utilization of ART in Ethiopia. This is based on the fact that a lot of PLwHA refused to take ART and stopped taking ART because of socio-economic and related reasons. Food stuff, such as ready-to-use supplementary food rations, has been provided based on the body mass index of the patient. The number of patients who received food and nutrition support increased from less than 43,000 to more than 140,000 between 2007/8 and 2011/12. Income generating activities, including training and seed money, have been given to PLwHA so that they will generate income by themselves. Home and community-based services, including psychosocial support, have also been provided to them [14].

### Patient retention in care

In spite of the achievements in scaling up ART in the country, losses to follow-up and early mortality of patients on ART have been challenges for the ART program. Only 65% of the patients were retained after two years on ART by 2008 [14]. Nevertheless, the ‘current retention’ rate (the retention rate during a specific *“calendar”* among patients who are on ART sometime during the *“calendar”)* had improved significantly from 77% (23% attrition) in 2004/5 to 92% (8% attrition) in 2012/13 [14] (Table 1).

**Table 1:** Current retention rate per year in the ART program in Ethiopia, 2004/5-2012/13

**Year**	**Currently on ART by the end of the year (a)**	**Attrition during the year (b)**	**Total (c)=a+b**	**Attrition rate (d)=b/c**	**Current retention rate (e)=1-d**
2004/5	18,594	5,642	24,236	23%	77%
2006/7	72,100	15,099	87,199	17%	83%
2008/9	153,741	17,371	171,112	10%	90%
2010/11	247,805	24,428	272,233	9%	91%
2012/13	308,860	25,959	334,819	8%	92%

The improvement in retention in care was possible as a result of a variety of interventions implemented in the country. The key informants indicated that a number of initiatives, including (1) case management program, (2) multi-disciplinary team and catchment area meetings, (3) patient information systems, and (4) provision of care and support services, (5) decentralization and task shifting, and (6) framework for linkage to care, were designed and implemented in the country to improve retention in care.

### Case management

The key informants emphasized that the “case management program” was one of the initiatives which had been in place in the country since 2007/8. They said:

“The ‘case management program’ is designed to improve the provision and coordination of activities by different actors in the continuum of care. The “case manager” is at the center of the coordination of care provided at the health facility and community levels. The “case manager” with his/her subordinates, expert patients and outreach workers, coordinates the services provided to the patients by the health facilities and community-based organizations.”

The key informant added:

“Defaulter tracing and outreach services were also provided by adherence supporters. These cadres went out from the health facility to the community and trace patients who didn’t show up for their appointment.”

### Multi-disciplinary team and catchment area meetings

The key informants have also added other related mechanisms to improve retention in care: “multi-disciplinary team meetings” and “catchment-area meetings”. They said:

“‘*Multi-disciplinary team meetings’ involve the different service providers, including clinicians, adherence counselors, pharmacists, case managers, adherence supporters and lab technologists, at health facility level. They discuss about patients who are either at risk of adherence and/or retention, or lost to follow-up or dead. These meetings are conducted regularly and attended by all the different providers in the health facility.”*

“‘Catchment area meetings’ are conducted among health facilities, located in one catchment area as defined by the administration, and community-based organizations. The participants discuss about patients who are lost to follow-up, dead, transfer in or transfer out within the catchment area. These meetings are conducted regularly and attended by program managers and service providers from all the health facilities and community-based organizations in the catchment area.”

### Patient information systems

Key informants have also identified “improved patient information system” as a key tool in improving patient retention in Ethiopia. They said:

“Patient information system is about the documentation, updating and sharing of the patient information related to residency, telephone, side-effects of drugs, socio-economic status, and outcomes. The data clerks are at the center of the patient information system in health facilities. When patients are registered for care they are requested to bring their identification card for their valid address. Moreover, patient’s address is updated whenever the patient comes for their drug refill. The data clerks identify patients “who should come when (every day, every week, today, tomorrow, next week, next month, and so on)” and “who didn’t come for refill when (yesterday, last week, last month, and so on)”. The data clerks then send the list of patients to clinicians and “case managers” for their respective actions.”

### Provision of care and support services

“Provision of care and support services for patients on ART” was also found to play a key role in improving retention in care. The key informants said that:

“Fear of stigma, long distance from health facilities, transport cost, feeling healthy and better, lack of nutrition and opting for traditional medicines are the main reasons for inadequate linkage to care and retention in care in Ethiopia. Hence, care and support services including home-based care, nutrition and financial support are instrumental in improving adherence and retention.”

### Decentralization and task shifting

Decentralization and task shifting were also mentioned to improve retention in care. Key informant said:

“Decentralization of the services has moved the treatment closer to patient's home and minimizes the indirect costs related to transportation and working hours. Task shifting was also said to decrease the waiting time to see the clinician when low-and mid-level health care providers are taking additional responsibilities and share the tasks of the physician.”

### Linkage to care

The key informants emphasized that:

“Early linkage to care and initiation on ART has played a role in improving retention in care in the country. Once linkage was identified as a challenge for the HTC and ART programs in the country, a strategic framework for improving linkage between testing and care facilities was developed. This has been implemented and helped to improve retention in care.”

## Discussion

Ethiopia has been able to scale up ART in spite of the limited resources available in the country. The number of health facilities providing ART has increased significantly and reached 913 in 2013. The number of PLwHA started on ART has also increased markedly from 9,000 in 2005/6 to more than 439,000 in 2012/13. Retention in care was recognized to be a challenge for the ART program. A number of interventions, including programmatic/health systems, structural, socio-cultural and health information systems, have been implemented to address this challenge. As a result, retention in care had been progressively improving; current retention rate per year increased from 77% in 2004/5 to 92% in 2012/13.

Health systems, societal and individual factors were identified to be barriers to access to ART in resource-limited countries [22]. In spite of these barriers enormous progress has been made in the last decade with the scale-up of ART in many low- and middle-income countries. A number of global initiatives as well as international recommendations, including the public health approach for ART delivery, have enabled these countries to scale up ART [23,24]. Decentralization and task shifting are the two pillars of the public health approach used to scale up ART [23].

Decentralization of ART delivery to primary health center level has increased access and utilization of ART in Ethiopia [12,16]. Decentralization of ART to rural health facilities appears to be an effective and safe way to scale up ART and improve equity in geographical access to ART [23,24]. Task shifting has been promoted widely as a mechanism for expanding access to ART [23,25]. In Ethiopia, task shifting to low- and mid-level health workers has enabled large scale expansion of ART [17]. Task shifting from doctors to nurses or from health care professionals to lay health workers can be implemented without compromising health outcomes for patients [25,26].

During the large scale expansion of ART in Ethiopia, retention in care has been a challenge. We found that a mix of factors including operational and programmatic (decentralization, task shifting and linkage), structural (distance to the clinic, transportation and poverty), socio-cultural (social support, stigma, disclosure, and traditional and religious beliefs), and patient information systems were associated with retention in care.

Decentralization was found to facilitate improved retention in care in Ethiopia. In a previous study in Ethiopia, it was also found that retention in care was better at decentralized primary health facility levels than at centralized hospital level [17,27]. In the district of Lusikisiki, South Africa, the rate of loss to follow-up among patients who started ART at decentralized sites was almost 10-fold lower than at centralized sites (2% vs. 19% lost to follow-up) [28]. In Malawi, it was demonstrated that a centralized ART clinic lost patients faster than a decentralized one [29]. Better retention was associated with presence of peer support groups (two times more) and outreach services (two times more) [30,31]. A study in rural KwaZulu-Natal, South Africa, found that outcomes of patients were not affected by decentralization of ART [32].

Task shifting, from doctors to nurses or from health care professionals to lay health workers, was recognized to facilitate ART scale up and retention in care. It was also found to be feasible and implemented without compromising health outcomes [25]. In Ethiopia, it was found that ART delivery by health officers and nurses did not compromise patient outcomes [17,27]. Systematic reviews show that task shifting is an effective and cost-effective strategy for ART rollout [33,34]. In a cluster-randomized trial conducted in South Africa, it was found that time to death did not differ between intervention (task shifting) and control (no task shifting) groups [35]. Similar results were found in Cameroon and Rwanda [36,37].

Early linkage from HIV testing to care was crucial for improving retention in care. In a previous study from Ethiopia, it was found that linkage to care was only 61% after HIV test results [10]. In a study from South Africa, only 63% of PLwHA visited a health facility for a CD4-cells count measurement within 6 months of testing HIV positive [38]. A systematic review shows a substantial loss of patients at every step, along the continuum of care, starting with patients who do not link to care [39]. Linkage to care allows provision of prophylaxis for opportunistic infections, regular monitoring and timely initiation of ART [40-44].

Distance and transportation to the ART clinic were found to be important factors to improve retention in care in the Ethiopian ART program. They are also found to be major barriers to retention in care in other resource-limited settings [9,10,45-48]. Similar results were found in rural Uganda, Malawi, Cambodia and India [49-52]. Finance (costs for food and shelter, in addition to the transport cost) was reported to be a bottleneck for linkage and retention in care in Ethiopia [45,46]. In South Africa and Uganda, around 35% of patients stated finance as a reason for absence from clinic visits [53,54]. In a randomized trial conducted in Uganda, around 18% of patients were lost among individuals who received a cash transfer for transportation while more than around 34% were lost among individuals who did not receive this cash transfer [55].

Socio-cultural factors such as social relations, disclosure and stigma are found to be important determinants of retention in care in resource-limited settings. In Ethiopia, stigma and preference for traditional medicine and “holy water” were cited as some of the reasons for loss to follow-up from ART clinics [45]. In Malawi, it was found that stigma led to non-retention in 25% of patients on ART [56]. In Uganda, it was found that 1.2% of patients discontinued ART because of a belief in spiritual healing [57]. Programs with home- and community-based services had a lower loss to follow-up rate (1-5%) compared to programs without these services (10-14%) [58-63].

Patient information systems were identified to be crucial for improving retention in care. They can play a pivotal role in improving retention in care [26]. An appropriate measurement of patient retention helps program managers to monitor progress systematically, identify bottlenecks, and design and implement operational and programmatic changes timely [64]. However, the current approach for measuring retention focuses on a particular clinic or health facility, not on a network of health facilities. This underestimates the level of retention in care, as there are patients who change from their original clinic to (an) other clinic(s) during their lifelong ART journey while they are reported as lost to follow-up [47,48,53,54,65]. Moreover, the currently available information systems are not designed around a continuum of care that should start at the time of HIV diagnosis. This would allow programs to track the real outcomes of PLwHA from HTC services to care and treatment services [39]. It is therefore important that ART programs take into consideration these issues when they are redesigning their information systems.

Our study shows that the ART program in Ethiopia has managed to expand vastly over the eight years since its launch in 2005. Moreover, the program has developed a capacity to learn and improve gradually. This would not have been possible without the fast expansion and comprehensive strengthening of the entire health system. This in turn has been complemented by and depended on community mobilization, strong partnership and government leadership.

This study has a number of strengths. Firstly, it is based on data from a real life situation of a large scale ART program. This facilitates the transfer of knowledge to programs with similar contexts. Secondly, it is based on a mixed-methods study (using both quantitative and qualitative methods). Thirdly, it looks at both quantity (ART scale up) and quality (retention in care) of the ART program. Fourthly, the study has a longer-term (eight years) and a broader (including health systems strengthening and community mobilization) scope to understand scaling up ART and retaining patients in care. The study will thus add value to knowledge and practice of ART as most of the available studies are with short-term and narrower views. The study also has a number of limitations. Firstly, the routine program data may lack quality because of poor documentation. However, the large number of health facilities and patients included in the study may compensate for this limitation. Secondly, the study didn’t assess the change in baseline characteristics of patients started on ART. It is likely that baseline characteristics, such as the mean CD4-cells count, have changed over time and contributed to the change in retention in care.

We recommend the following for future research: (1) How to sustain the current successes in the long-term. The country has been able to implement a variety of interventions to improve retention in care. Nevertheless, it is important to sustain these gains in the long-term. Therefore, it is important that a study is conducted to identify how the country can sustain the successes achieved so far. (2) Feasibility, acceptability and effectiveness of using the health extension program (HEP) for improving retention in care needs to be studied. The HEP can be a very good opportunity to sustain the gains so far. (3) The change in the baseline characteristics of patients started on ART overtime. This is an important limitation of our study. This helps to understand the link between the change in the baseline characteristics of patients started on ART and the change in retention in care overtime.

## Conclusion

Ethiopia has been able to scale up ART in spite of the limited resources available in the country. This has been possible due to different initiatives including the public health approach, health systems strengthening, community mobilization and provision of care and support services. While ART was being scaled up in Ethiopia, retention in care was later recognized as a real challenge for the program. The program has thus tried to identify and implement interventions to improve retention in care. A number of initiatives, including case management program, catchment area meetings, patient information systems, provision of care and support services, decentralization and task shifting, and framework for linkage to care, were designed and implemented to improve retention in care. Hence, we recommend that models of care for scaling up ART and improving retention in care should take into account these factors.

## Competing interests

The authors declare that they have no competing interests.

## Authors’ contributions

YA: conceived the study, coordinated and participated in the data collection, conducted the data analysis and interpretation, developed the first draft, and revised subsequent drafts. AA: commented on successive drafts. ML: commented on successive drafts. LL: commented on successive drafts. EW: commented on successive drafts. WVD: advised on the data analysis and interpretation, commented on successive drafts. All authors approved the final version for submission.
